# Prevalence of abnormal glucose metabolism among adults attending an outpatient department at a tertiary referral hospital in Swaziland: a cross-sectional study

**DOI:** 10.1186/s12889-020-08489-9

**Published:** 2020-03-26

**Authors:** Mojeed Akorede Gbadamosi, Boikhutso Tlou

**Affiliations:** Discipline of Public Health Medicine, School of Nursing and Public Health, University of KwaZulu-Natal, Durban, South Africa

**Keywords:** Abnormal glucose metabolism, Diabetes, Pre-diabetes, Prevalence, Swaziland

## Abstract

**Background:**

The exact prevalence of type 2 diabetes mellitus (T2DM) and pre-diabetes in Swaziland remains unknown. Estimates suggest that the prevalence rate of type 2 diabetes mellitus is between 2.5 and 6.0% in Swaziland. The disparity in these estimates is due to a lack of quality data but the prevalence of diabetes is increasing in Swaziland. This study estimates the prevalence of type 2 diabetes mellitus and pre-diabetes among patients in a tertiary hospital in Manzini, Swaziland.

**Methods:**

A cross-sectional observational survey was used to estimate the crude and age-adjusted prevalence rates of diabetes and pre-diabetes (impaired fasting glucose (IFG) and impaired glucose tolerance (IGT)) in the Manzini regional referral hospital of Swaziland. Diabetes was defined as a fasting blood glucose (FBG) ≥ 7.0 mmol/L (126 mg/dL) and pre-diabetes was defined as an FBG of 6.1–6.9 mmol/L (110–125 mg/dL) and an FBG < 7.0 mmol/L (< 126 mg/dL), respectively for IFG and IGT. A random sample of 385 participants was used. Data analysis was done using SPSS version 26 and the level of statistical significance was set at α < 0.05.

**Results:**

The crude prevalence of type 2 diabetes mellitus and pre-diabetes was 7.3% [95% CI 4.9–10.3] and 6.5% [95% CI 4.2–9.4], respectively, with clear gender differences in the prevalence of diabetes (men 1.6% vs women 5.7%, *p* = 0.001). On the other hand, significantly more men (3.6%) had pre-diabetes than women (2.9%) (*p* = 0.004). The overall age-adjusted prevalence rates of type 2 diabetes mellitus and pre-diabetes were 3.9 and 3.8%, respectively. Among the diabetic group, 3 (10.7%) had known T2DM, whereas 25 (89.3%) were newly diagnosed during the study. Advancing age, gender, raised blood pressure, abnormal body mass index, and wealth index were significant risk factors for T2DM or prediabetes.

**Conclusion:**

The prevalence of type 2 diabetes mellitus among adult outpatients in the Raleigh Fitkin Memorial hospital was higher than previously reported in the health facility in Manzini; suggesting the need for routine T2DM screening at outpatient departments.

## Background

The exact prevalence of type 2 diabetes mellitus (T2DM) in Swaziland remains unknown. The International Diabetes Federation (IDF) [1] reported that the proportion of adults living with T2DM in Swaziland was 2.5%. On the contrary, the World Health Organisation (WHO) [2] estimated the prevalence of T2DM in Swaziland to be 6%. Both the IDF and WHO extrapolated data from a similar country to estimate the T2DM prevalence for Swaziland, due to absence of original data from Swaziland. Furthermore, a recent Stepwise approach to surveillance (STEPS) survey conducted in 2014 highlighted the significance of the burden of chronic diseases of lifestyle in Swaziland [3]. The survey findings revealed that about 14.2% of the respondents had raised blood glucose, while 9.8% had impaired fasting glycemia (IFG).

The STEPS survey [3] attributed the rising prevalence of chronic diseases (including diabetes mellitus) to the high rates of obesity, physical inactivity, smoking, and alcohol consumption in the Swazi population. Therefore, the Swaziland government established the National Non-Communicable Diseases Programme with a focus on cardiovascular diseases, diabetes, cancer, and other chronic diseases [4]. Furthermore, the Ministry of Health (MOH) developed the National Non-Communicable Diseases Policy [5] and a strategic plan for 2012–2017 [6] to guide the implementation of non-communicable diseases (NCD) prevention, management, and control. There is thus a serious need for current epidemiological data.

Furthermore, about 8.8% of the STEPS respondents aged 40–69 years had cardiovascular disease [3]. The morbidity and mortality statistics for chronic diseases showed alarming rates [3]. The Swaziland Ministry of Health (MOH) [4] also reported that diabetes mellitus was responsible for 73,290 attendances at out-patient departments of all health facilities in 2016, representing a 16.8% increase from the 2015 figures. Similarly, mortality due to diabetes mellitus was unacceptably high and has increased sharply from 45 cases in 2014, to 88 in 2015, and finally to 109 cases in 2016 [4].

To the researchers’ knowledge, no epidemiological study has been conducted in Swaziland to estimate the age-adjusted prevalence of T2DM and pre-diabetes, even though diabetes ranked third among the ten leading causes of all hospital admissions in Swaziland in 2016 [4]. Previous studies in Swaziland have investigated the influence of diabetes on the quality of life [7] as well as the prevalence of diabetic retinopathy [8], and a case has been made for strengthening chronic care services in the kingdom [9]. Similarly, risk factors for non-communicable diseases (NCDs) in Swaziland have been reported [3]. A study by Rabkin et al. in 2016 investigated the possibility of screening for diabetes among HIV-positive patients [10]. However, the study did not report age-adjusted prevalence rates for pre-diabetes and diabetes. To address these gaps, the current study was conducted to determine the prevalence of T2DM and pre-diabetes in a tertiary hospital in the Manzini region of Swaziland. Quantifying the prevalence of T2DM and pre-diabetes among patients attending the out-patient departments (OPD) at Raleigh Fitkin Memorial (RFM) will contribute to the understanding of the burden of T2DM in Swaziland. Findings from this study will facilitate planning and implementation of cost-effective interventions for the prevention and management of diabetes mellitus, by adding new scientific information to the existing body of knowledge.

## Methods

### Study design

This study was an observational, cross-sectional, hospital-based study.

### Study setting

Raleigh Fitkin Memorial (RFM) Hospital, situated in the Manzini region, is the second largest public health facility in Swaziland. It is a regional referral and government sub-vented teaching hospital. The mission hospital and its different service units draw patients from the Manzini region as well as neighbouring communities in the adjacent regions including the Mbabane – Manzini – Siteki corridor.

### Population and sample

A cross-sectional study was conducted from February to March 2019 among 385 patients who visited the OPD of the RFM Hospital in Manzini, Swaziland. All patients who visited the OPD during the data collection period were included. Patients were included in the sample if they resided in the Manzini region; were 18 years of age and above; had fasted for 8 h before the screening, and willingly consented to participate in the study. However, patients with active infection or using corticosteroids; who were pregnant; who had a temperature greater than 101.4 °F (38.6 °C); were taking antibiotics or anti-malaria medications or who were unwilling to participate were excluded. A representative sample of the medical and surgical outpatient population was surveyed. A systematic random sampling technique was applied to sample 385 individuals, whereby every 3rd individual visiting the OPD was approached to participate in the study, bearing in mind the inclusion criteria. As remarked by Castillo [11], systematic random sampling allows the researchers to add a degree of a system into the random selection of subjects and ensures that the population is evenly sampled.

The sample size was estimated with the formula for a cross-sectional study by Daniel [12]:

$$ \mathrm{n}=\kern0.37em \frac{{\mathrm{Z}}^2\left(\mathrm{PQ}\right)}{\updelta^2} $$

Z = 1.96. (The standard normal deviation at 95% confidence)

*P* = 0.5 (Estimated prevalence of the problem under study, assumed to be 50% to achieve the maximum sample size)

Q = 0.5 ((100% - P) (or 1-P))

δ = 0.05 (The precision or maximum acceptable error)

n = Sample size

$$ \mathrm{n}=\frac{(1.96)^2\times (0.5)(0.5)}{(0.05)^2} $$

n = 384.16

The sample size was 385 patients reporting to the Medical and Surgical out-patient departments of the Raleigh Fitkin Memorial Hospital. This method of sampling has some selection bias but it is easier to perform and less time consuming.

### Definitions

The prevalence of T2DM was defined to comprise of all participants with known diabetes and those with a fasting blood glucose (FBG) ≥ 7.0 mmol/L (126 mg/dL) or a random blood glucose (RBG) ≥ 11.1 mmol/dL (> 200 mg/dL) and/or an oral glucose tolerance test (OGTT) ≥ 11.1 mmol/dL (> 200 mg/dL). Prediabetes was defined as a FBG between 6.1 and 6.9 mmol/L (110–125 mg/dL) for impaired fasting glucose (IFG) or an OGTT between 7.8 and 11.0 mmol/L (140–199 mg/dL) for impaired glucose tolerance (IGT) based on the WHO/IDF recommendation [13]. Abnormal glucose metabolism (AGM) was defined as IFG, IGT, or T2DM.

### Measures

#### Demographics

Demographic variables collected included age; gender; marital status; and residential location.

#### Socio-economic status

Participants were asked to report their level of education, categorised into five groups: no formal education; primary school; secondary school; high school; and tertiary education. Respondents’ occupations and average monthly household incomes were also determined. Household wealth (assets, ownership of agricultural land and farm animals) was also recorded and used to generate wealth quintiles through principal component analysis (PCA). Principal component analysis was used to extract a set of uncorrelated principal components as weighted linear combinations of the original household asset ownership variables. Each principal component was the sum of each variable multiplied by its weight. The components were ordered so that the first principal component explained the largest amount of variation in the data. Therefore, the first principal component was used to represent the wealth index because it explained the largest possible amount of variation in the original data [14]. This wealth index was then ranked into five quintiles (since the wealth index was a continuous variable), dividing all participants into five equal groups; lowest, second, third, fourth, and the highest quintiles.

#### Anthropometric variables

Weight was measured in kilograms (kg) using a weighing scale (FORA Digital Multi-function Diamond Scale), and height was measured in centimeters (cm) using a stadiometer. Weight was measured with the participants wearing light clothing and barefoot. Waist and hip circumferences were assessed in centimeters (cm) using a measuring tape. Waist and hip circumferences were assessed while the participants were in a standing position.

The Body mass index (BMI) was calculated as weight divided by height squared (kg/m^2^). Participants were classified as underweight if their BMI was < 18.5 kg/m^2^, normal weight if their BMI was between 18.5 and 24.9 kg/m^2^ (inclusive), overweight if their BMI was between 25.0 and 29.9 kg/m^2^ (inclusive) and obese if their BMI was ≥30 kg/m^2^ [15].

A raised waist circumference was defined as a waist circumference greater than 94 cm (> 94 cm) for men, and greater than 80 cm (> 80 cm) for women, while a raised waist-to-hip ratio (WHR) was defined as a WHR greater than one (> 1.0) for men and greater than zero point eight five (> 0.85) for women, according to standardised international criteria [16].

#### Blood pressure

Blood pressure was measured using a sphygmomanometer (FORA Fully Auto Desk P30 Plus Spygmo Digital with 24 – 43 cm WIDR Cuff). The blood Pressure measurement was taken while the participant was in the sitting position, using the right upper arm after a 5-min rest period. Two readings were taken during the interview at 15 min intervals.

Participants were hypertensive (stage 1) if their mean systolic blood pressure (SBP) was 130–139 mmHg or their mean diastolic blood pressure (DBP) was 80-89  mmHg. Stage 2 hypertension was defined as a mean SBP of 140–179 mmHg and a DBP greater than or equal to 90  mmHg. Participants were considered to have hypertensive crisis if their mean SBP was greater than or equal to 180 mmHg or if their mean DBP was greater than or equal to 120 mmHg. A mean SBP of 120–129 and a mean DBP of less than 80 mmHg was considered as an elevated blood pressure. Participants on anti-hypertensive treatment were also included. Hypertension definitions were made according to the American College of Cardiology/American Heart Association guidelines [17].

#### Blood glucose

The fasting blood glucose (FBG) and Oral Glucose Tolerance (OGT) testing were done using a glucometer (On Call® EZ II, ACON Laboratory, Inc., USA) according to the manufacturer’s instructions. Fasting blood glucose was measured between 08:00 and 12:00, after a minimum of 8 h of overnight fasting. To determine the Oral Glucose Tolerance (OGT), 75 g of glucose (Dextrose Monohydrate, MEDICOLAB, South Africa) was dissolved in 300 ml of water and given to the respondent to drink within 5 min after a taking an FBG reading. After a two- hour interval, the glucose test was performed again.

### Prevalence of pre-diabetes and type 2 diabetes mellitus

The calculation of the prevalence of T2DM and pre-diabetes was based on the method used by Miller [18]. The number of cases of T2DM or pre-diabetes was divided by the sample size to obtain the crude prevalence of T2DM or pre-diabetes, respectively. The age-adjusted prevalence rates were calculated by dividing the number of cases of T2DM or pre-diabetes, respectively in each of the 10-year age groups by the number of cases in that group, before the result was multiplied by the population proportion for that age group, based on the Swaziland Demographic and Health Survey (DHS) report [19]. (See Supplementary file 1).

### Statistical analysis

All analyses were conducted using SPSS version 26 statistical software (IBM Corp., Armonk, NY, USA). Descriptive statistics (percentage) was used to describe the crude (unadjusted) prevalence rates for the sample and age-adjusted prevalence rates of T2DM and pre-diabetes. A Chi-square test was applied to examine the association between the independent demographic and biometric variables and abnormal glucose metabolism (AGM) (T2DM and pre-diabetes). Analysis of variance (ANOVA) was used to examine the group and the main effect of the categorical independent variables (age groups, gender) on the continuous dependent fasting blood glucose (FBG). A two-sided *p*-value < 0.05 was considered statistically significant.

## Results

### Description of the study population

Four hundred and eleven (411) subjects were interviewed among the 440 subjects invited to participate, yielding a response rate of 93.4%. Of the 440 subjects invited, a total of 55 subjects were excluded due to being younger than 18 years of age (13 subjects), being resident outside of Manzini region (27 subjects), pregnancy (2 subjects), and high temperature (3 subjects). An individual withdrew for fear of the needle prick. Completed fasting blood glucose readings were available for 394 subjects but 9 subjects had completed questionnaires with missing data.

Table 1 shows the characteristics of the study participants. There were 197 (51.2%) men and 188 (48.8%) women. A typical participant in this study was 38 years of age, with women being older than men (39 vs 36 years). The participants were evenly distributed in terms of areas of residence, with 50.1% (*n* = 192) and 49.9% (*n* = 191) from the rural and urban areas, respectively. A significantly higher proportion of women compared to men were overweight (33.5% vs 19.8%) and obese (33.5% vs 6.6%), unemployed (32.4% vs 21.8%), and from poor households (35.1% vs 20.8%). More men were significantly underweight (9.6%) than women (1.1%). Also, more men attained high school or college education than women (48.7% vs 34.0%). The mean systolic and diastolic blood pressures were 123.13 (16.22) mmHg and 77.83 (11.33) mmHg, respectively. According to the American College of Cardiology/American Heart Association’s (ACC/AHA) definition of hypertension [17], 36.9% of the study participants had normal blood pressure, 14.8% of them had pre-hypertension and 29.4 and 17.4% had stage 1 and 2 hypertensions, respectively, while six participants (1.6%) had hypertensive crisis. There was no significant gender difference in the prevalence of hypertension.

**Table 1:** Description of the study participants

Characteristics	Total	Men	Women	***p***-value
**Biomedical**^**a**^				
Sample size, n (%)	385 (100.0)	197 (51.2)	188 (48.8)	
Age (years), mean (SD)	37.77 (15.66)	36.47 (15.63)	39 (15.61)	0.094
BMI (kg/m^2^), mean (SD)	25.67 (5.97)	23.17 (4.11)	28.28 (6.49)	< 0.0001**
Hip circumference (cm), mean (SD)	100.09 (13.83)	95.02 (1.96)	105.40 (13.68)	< 0.0001**
Weight (cm), mean (SD)	70.87 (15.16)	63.31 (12.74)	73.56 (16.97)	0.001**
Blood Glucose (mmol/L), mean (SD)				
Fasting Blood Glucose	5.385 (1.211)	5.286 (0.888)	5.478 (1.449)	0.141
Random Blood Glucose	7.368 (1.830)	7.117 (1.632)	8.055 (2.235)	0.149
BP (mm Hg), mean (SD)				
SBP	123.13 (16.22)	122.64 (13.99)	123.64 (18.29)	0.549
DBP	77.83 (11.33)	77.90 (10.57)	77.77 (12.11)	0.909
BMI (kg/m^2^), n (%)				< 0.0001*
Underweight	21 (5.5)	19 (9.6)	2 (1.1)	
Normal	186 (48.3)	126 (64.0)	60 (31.9)	
Overweight	102 (26.5)	39 (19.8)	63 (33.5)	
Obese	76 (19.7)	13 (6.6)	63 (33.5)	
Waist circumference (cm), n (%)				0.855
Normal	221 (57.4)	112 (56.9)	109 (58.0)	
Overweight	59 (15.3)	29 (14.7)	30 (16.0)	
Obese	105 (27.3)	56 (28.4)	49 (26.1)	
Waist-to-hip ratio, n (%)				0.640
Normal	247 (64.2)	129 (65.5)	118 (62.8)	
Overweight	49 (12.7)	22 (11.2)	27 (14.4)	
Obese	43 (23.1)	46 (23.4)	43 (22.9)	
Hypertension (mmHg), n (%)				0.198
Normal	142 (36.9)	68 (34.5)	74 (39.4)	
Elevated	57 (14.8)	33 (16.8)	24 (12.8)	
Hypertension stage 1	113 (29.4)	63 (32.0)	50 (26.6)	
Hypertension stage 2	67 (17.4)	32 (16.2)	35 (18.6)	
Hypertension crisis	6 (1.6)	1 (0.5)	5 (2.7)	
**Socioeconomic**^**b**^				
Education, n (%)				0.054
No formal education	26 (6.8)	11 (5.6)	15 (8.0)	
Primary	96 (24.9)	45 (22.8)	51 (27.1)	
Secondary	101 (26.2)	44 (22.3)	57 (30.3)	
High school	118 (30.6)	67 (34.0)	51 (27.1)	
College/university degree	42 (10.9)	29 (14.7)	13 (6.9)	
Postgraduate degree	2 (0.5)	1 (0.5)	1 (0.5)	
Occupation, n (%)				0.022*
Student	49 (12.7)	28 (14.2)	21 (11.2)	
Unemployed	104 (27.0)	43 (21.8)	61 (32.4)	
Civil servant	157 (40.8)	91 (46.2)	66 (35.1)	
Self employed	58 (15.1)	30 (15.2)	28 (14.9)	
Domestic worker	8 (2.1)	1 (0.5)	7 (3.7)	
Shop attendant	9 (2.3)	4 (2.1)	5 (2.7)	
Residence, n (%)				0.331
Rural	192 (50.1)	94 (47.7)	98 (57.2)	
Urban	191 (49.9)	103 (52.3)	88 (47.3)	
Marital status, n (%)				0.081
Single	172 (44.8)	99 (50.3)	73 (39.0)	
Married	157 (40.9)	74 (37.6)	83 (44.4)	
Staying with partner	35 (9.1)	18 (9.1)	17 (9.1)	
Widowed	14 (3.6)	5 (2.5)	9 (4.8)	
Divorced	6 (1.6)	1 (0.5)	5 (2.7)	
Average monthly income (SDE), n (%)				0.001*
< 500	41 (10.6)	18 (9.1)	23 (12.2)	
500–999	66 (17.1)	23 (11.7)	43 (22.9)	
1000–1999	92 (23.9)	41 (20.8)	51 (27.1)	
2000–2999	74 (19.2)	42 (21.3)	32 (17.0)	
≥ 3000	112 (29.1)	73 (37.1)	39 (20.7)	

^ab^Mean with SD for biomedical data and percentages for socio-demographic data

*Chi-square or Fisher’s test, *p* < 0.05 was considered significant for men vs women

**Univariate ANOVA, *p* < 0.05 was considered significant for gender differences between men and women

BMI, Body mass index; BP, Blood pressure; DBP, Diastolic blood pressure; SBP, Systolic blood pressure; SD, Standard deviation; SDE: Swazi Emalangeni; n, Sample size

### Prevalence of pre-diabetes and type 2 diabetes mellitus

The overall prevalence rates of T2DM and pre-diabetes were 7.3 and 6.5%, respectively. The prevalence of T2DM was higher in women (3.6%) than men (2.9%). On the contrary, the prevalence of pre-diabetes was higher in men (5.7%) compared to women (1.6%). Based on the Swaziland national population estimates [19], the age-adjusted prevalence rates for T2DM and pre-diabetes were 3.9% (1.2% men vs 6.6% women) and 3.8% (4.1% men vs 3.6% women). The pre-existing T2DM was reported by three participants (10.7%) while the proportion of newly diagnosed T2DM among T2DM cases was 89.3% (Table 2).

**Table 2:** Distribution of abnormal glucose metabolism according to age and gender

				Crude Prevalence						Age-adjusted Prevalence*					
	Total			Proportion of pre-diabetes			Proportion of T2DM			Proportion of pre-diabetes			Proportion of T2DM		
Age group (years)	All	M	W	All	M	W	All	M	W	All	M	W	All	M	W
15–24	91	49	42	1.0	0.5	0.5	1.0	0.0	1.0	1.0	0.9	1.0	1.0	0.0	2.0
25–34	108	63	45	1.3	1.3	0.0	1.3	0.5	0.8	0.6	0.9	0.0	0.6	0.4	0.8
35–44	84	40	44	0.3	0.0	0.2	1.8	0.5	1.3	0.1	0.0	0.2	0.7	0.4	1.0
45–54	40	13	27	1.6	1.0	0.5	1.3	0.0	1.3	0.9	1.6	0.5	0.8	0.0	1.3
55–64	31	17	14	1.8	0.8	1.0	0.8	0.3	0.5	1.0	0.7	1.3	0.4	0.2	0.7
65+	31	15	16	0.5	0.0	0.5	1.0	0.3	0.8	0.3	0.0	0.6	0.5	0.2	0.9
**Total**	**385**	**197**	**188**	**6.5**	**3.6**	**2.7**	**7.3**	**1.6**	**5.7**	**3.8**	**4.1**	**3.6**	**3.9**	**1.2**	**6.6**

M, men; W, women

*Based on the Swaziland Demographic and Health Survey 2006/07 [19] (See supplementary Table 1)

Mean values of the fasting blood glucose (FBG) within age groups, hypertension, body mass index (BMI), waist circumference (WC) and waist-to-hip ratio (WHR) are shown in Table 3. Considering the categories within the variables, the concentration of FBG significantly increased with advancing age and BMI. The highest blood glucose concentration among women was observed within the age group of 55–64 years and within 45–54 years of age among men. Meanwhile, overweight and obese women (by BMI) had significantly higher blood glucose concentration levels than their counterparts of overweight and obese men (*p* < 0.0001). The blood glucose concentration levels were higher among hypertensive women than hypertensive men. Similar outcomes were obtained for gender distribution of those overweight and obese (by WC). However, the blood glucose concentration level was higher for obese men than that of obese women (by WHR).

**Table 3:** Mean glucose concentrations (mmol/L) according to gender

Variables	Men	Women	Total	***P***-value
Sample, n (%)	167 (48.5)	177 (51.5)	344 (100.0)	
Age groups (years), mean (SD)				< 0.0001**
15–24	4.989 (0.7534)*	5.154 (1.1734)*	5.066 (0.9708)	
25–34	5.275 (0.6706)	5.152 (0.8303)	5.222 (0.7424)	
35–44	5.260 (0.6162)	5.395 (1.3199)	5.337 (1.0118)	
45–54	5.500 (0.8234)	5.958 (2.0777)	5.813 (1.7790)	
55–64	5.693 (0.7918)	5.879 (1.2540)	5.786 (1.0334)	
65+	5.800 (2.0356)	6.200 (2.1147)	6.029 (2.0526)	
Hypertension, mean (SD)				0.175
Normal	5.277 (1.1124)	5.277 (1.4334)	5.277 (1.2903)	
Elevated (Prehypertension)	5.281 (0.7547)	5.187 (0.7724)	5.241 (0.7565)	
Hypertension stage 1	5.114 (0.6338)	5.778 (1.5632)	5.436 (1.2191)	
Hypertension stage 2	5.622 (0.8078)	5.661 (1.5967)	5.645 (1.3189)	
Body mass index, mean (SD)				< 0.0001**
Underweight	5.073 (0.7005)*	4.450 (0.2121)	5.012 (0.6800)	
Normal	5.164 (0.7260)	5.297 (1.4047)	5.211 (1.0182)	
Overweight	5.691 (1.2969)	5.809 (0.8469)	5.765 (1.6582)	
Obese	5.454 (0.6741)	5.363 (0.9437)	5.379 (0.8976)	
Waist circumference, mean (SD)				0.706
Normal	5.237 (0.7175)	5.426 (1.1279)	5.337 (0.9589)	
Overweight	5.254 (1.6173)	5.557 (1.6577)	5.422 (1.6315)	
Obese	5.385 (0.6804)	5.542 (1.9101)	5.458 (1.3872)	
Waist-to-hip ratio, mean (SD)				0.850
Normal	5.219 (0.7566)	5.493 (1.1921)	5.358 (1.0084)	
Overweight	5.319 (1.6500)	5.563 (1.7425)	5.456 (1.6891)	
Obese	5.451 (0.6099)	5.380 (1.8600)	5.415 (1.3829)	

Hypertension is defined as normal (SBP < 120 and DBP < 80 mmHg), Elevated (SBP 120–129 and DBP < 80 mmHg); hypertension stage 1 (SBP 130–139 or DBP 80–89 mmHg); hypertension stage 2 (SBP ≥ 140 or DBP ≥ 90 mmHg)

BMI defined as underweight (< 18.5), normal (18.5–24.9), overweight (25.0–29.9) and obese (≥ 30)

Waist circumference is defined as normal, overweight and obese for men (< 94, 94–102 and ≥ 103 cm) and women (≤ 80, 80–88, and ≥ 89 cm), respectively

Waist-to-hip ratio is defined as normal, overweight and obese for men (≤ 0.95, 0.96–1.0 and ≥ 1.1) and women (≤ 0.80, 0.81–0.85, and ≥ 0.86), respectively

n, number; *p < 0.05 and **p < 0.05 significant by Univariate ANOVA (F-test) for gender difference in subgroup and group, respectively

### Factors associated with pre-diabetes and type 2 diabetes mellitus

#### Factors associated with pre-diabetes

Table 4 shows the findings of chi-square analysis to identify the association between socioeconomic and demographic variables and the presence of diabetes and pre-diabetes (AGM). Individuals’ age, raised blood pressure, and excess body weight were found to be significantly associated with pre-diabetes. Over half of the subjects with pre-diabetes were in the age group 45 years and older. The majority of the subjects with abnormal glucose were hypertensive, overweight or obese.

**Table 4:** Demographic and socio-economic factors associated with diabetes status

Variable	NGM^**b**^	AGM^**a**^			
		Prediabetes	***p***-value	T2DM	***p***-value
Sample, n (%)	332 (86.2)	25 (6.5)		28 (7.3)^c^	
Mean age (SD)	36.58 (15.01)	45.68 (17.35)		44.82 (18.5)	
Age (years) (n, %)			0.001*		0.255
15–24	83 (25.0)	4 (6.0)		4 (14.3)	
25–34	98 (29.5)	5 (20.0)		5 (17.9)	
35–44	76 (22.9)	1 (4.0)		7 (25.0)	
45–54	29 (8.7)	6 (24.0)		5 (17.9)	
55–64	21 (6.3)	7 (28.0)		3 (10.7)	
65+	25 (7.5)	2 (8.0)		4 (14.3)	
Gender (n, %)			0.795		0.001*
Men	177 (53.3)	14 (56.0)		6 (21.4)	
Women	155 (46.7)	11 (44.0)		22 (78.6)	
Residence (n, %)			0.563		0.717
Rural	165 (50.0)	14 (56.0)		13 (46.4)	
Urban	165 (50.0)	11 (44.0)		15 (53.6)	
Marital status (n, %)			0.131		0.371
Not in union^d^	171 (51.7)	9 (36.0)		12 (42.9)	
In union^e^	160 (48.3)	16 (64.0)		16 (57.1)	
Hypertension (n, %)			0.046*		0.710
Normal	127 (38.3)	4 (16.0)		11 (38.3)	
Elevated	51 (15.4)	3 (12.0)		3 (10.7)	
Stage 1	97 (29.2)	9 (36.0)		7 (25.0)	
Stage 2	57 (17.2)	9 (36.0)		7 (25.0)	
Body mass index (n, %)			0.042*		0.052
Underweight	20 (6.0)	1 (4.0)		0 (0.0)	
Normal	170 (51.2)	6 (24.0)		10 (35.7)	
Overweight	80 (24.1)	10 (40.0)		12 (42.9)	
Obese	62 (18.7)	8 (32.0)		6 (21.4)	
Waist circumference (n, %)			0.155		0.543
Normal	189 (56.9)	19 (76.0)		13 (46.4)	
Overweight	52 (15.7)	2 (8.0)		5 (17.9)	
Obese	91 (27.4)	4 (16.0)		10 (35.7)	
Waist-to-hip ratio (n, %)			0.025*		0.221
Normal	210 (63.3)	22 (88.0)		15 (53.6)	
Overweight	41 (12.3)	1 (4.0)		7 (25.0)	
Obese	81 (24.4)	2 (8.0)		6 (21.4)	
Education (n, %)			0.154		0.087
No formal education	19 (5.7)	4 (16.0)		3 (10.7)	
Primary	78 (23.5)	7 (28.0)		11 (39.3)	
Secondary or higher	235 (70.8)	14 (56.0)		14 (50.0)	
Occupation (n, %)			0.596		0.069
Student/unemployed	129 (38.9)	10 (40.0)		14 (50.0)	
Self-employed	49 (14.8)	2 (8.0)		7 (25.0)	
Salaried job	154 (46.4)	13 (52.0)		7 (25.0)	
Household income (SDE) (n, %)			0.977		0.065
< 999	87 (26.2)	7 (28.0)		13 (46.4)	
1000–2999	147 (44.3)	11 (44.0)		8 (28.6)	
≥ 3000	98 (29.5)	7 (28.0)		7 (25.0)	
Wealth index (n, %)			0.480		0.003*
Poorest	58 (17.5)	6 (24.0)		11 (39.3)	
Second	72 (21.7)	2 (8.0)		5 (17.9)	
Middle	64 (19.3)	6 (24.0)		0 (0.0)	
Fourth	64 (19.3)	5 (20.0)		5 (17.9)	
Richest	74 (22.3)	6 (24.0)		7 (25.0)	

^a^AGM, abnormal glucose metabolism; ^b^NGM, normal glucose metabolism; ^c^contains 3 (10.7%) previously diagnosed cases; ^d^includes single, widow, staying with partner;

^e^includes married, staying with partner; n, number; *chi-square statistic significant at *p* < 0.05

#### Factors associated with T2DM

Gender and wealth index were the non-modifiable and modifiable risk factors found to be associated with T2DM. A significant proportion of women compared to men were found to have T2DM (78.6% vs 21.4%). Moreover, subjects with T2DM were found to be significantly poorer than subjects with normal glucose metabolism (39.3% vs 17.5%). Body mass index, education, occupation and household income were also found to be associated with T2DM, but these did not reach the level of statistical significance (*p* > 0.05).

## Discussion

In this study the researchers reported estimates for the prevalence of T2DM and pre-diabetes, and the factors associated with these conditions among adults (18–83 years old) attending outpatient departments at the RFM in Swaziland. The overall prevalence of T2DM and pre-diabetes was 7.3 and 6.5%, respectively, with clear gender differences. The results showed a higher prevalence of T2DM (7.3%) than previously reported (5%) in this hospital. Similarly, the proportion of newly detected T2DM cases (89.3%) was high, suggesting that screening practices in this hospital were ineffective. Advancing age, female gender, excess body weight, poorest wealth index and high blood pressure were associated with a higher risk of T2DM and pre-diabetes in these Swazi adults.

The estimated T2DM prevalence of 7.3% found in the current study was higher than the 5.0% reported previously by Rabkin et al. [10] in the same setting, but lower than the estimate reported in the 2014 STEPS survey [3] in Swaziland. The difference between Rabkin et al.’s estimate and the current study could be due to differences in the diagnostic criteria used. As pointed out by the authors, glycated hemoglobin may have underestimated the prevalence of T2DM since the subjects were HIV-infected. Similarly, the possible differences in the STEPS survey’s estimate and the current study may have been due to the use of higher cut-off points in the current study. In the STEPS survey, T2DM was defined as a blood glucose reading of ≥6.1 mmol/L compared to a blood glucose reading of ≥7.0 mmol/L used in the current study.

This study’s reported prevalence rate for T2DM was much higher than those reported in many studies in the Southern African sub-continent [20–22], but lower than those found in some studies in South Africa by Werfalli et al. [23] and Oni et al. [24]. The difference between the T2DM prevalence rate in the current study and estimate by Werfalli et al. could have been due differences in participants’ age groups (older adults ≥50 years) and the use of self-report diagnosis instead of screening and diagnosis. It is known that the prevalence of T2DM peaks at around age 50 years [25], therefore, a higher prevalence estimate in Werfalli et al.’s study was expected. Similarly, the study by Oni et al. [24] used different diagnostic criteria (glycated haemoglobin (HbA1c)) compared to the current study which used fasting blood glucose (FBG). Oni et al. reported a lower prevalence rate for T2DM (4.1%) when diagnosed with FBG [24].

The high prevalence of newly diagnosed T2DM (89.3%) among patients found to have T2DM in the current study was consistent with the previous report in Swaziland [3] and evidence from South Africa [26], but higher than those (25–56.8%) reported in SSA [20, 21, 24, 27]. The implication of the high rate of newly diagnosed T2DM among T2DM cases was that without this study, these diabetic cases may not have sought health care for their condition until complications had set in. Moreover, the higher prevalence of newly detected cases of T2DM suggested that current screening practices in this hospital and SSA were ineffective [28]. Therefore, screening of high-risk individuals should be incorporated in the clinical practice at outpatient departments in this hospital.

The prevalence of pre-diabetes estimated in the current study (6.5%) was higher than the rates reported elsewhere in SSA: Kenya [29]; Malawi [30]; Uganda [31]; and South Africa [32]. Pre-diabetes is an intermediate metabolic state between normal glucose metabolism and T2DM, which are higher than normal glucose level and could progress to T2DM [13]. An IDF publication [33] estimated the prevalence of IGT to vary between 2.2 and 16% in Africa and asserted that the burden of T2DM would continue to escalate if nothing was done to curb the epidemy of IGT. It is therefore vital that these-at-risk groups be targeted for lifestyle changes to prevent full-blown T2DM in these individuals.

As expected, pre-diabetes was significantly associated with advancing age, consistent with previous reports in SSA [34–36]. However, a study in rural Uganda did not find an association between BMI and AGM [37], probably due to the younger age groups included (≥13 years) in their sample and the use of random blood glucose instead of fasting blood glucose results. In the current study, the proportion of obese women was significantly higher than the proportion of obese men. A possible explanation for this finding is that women are known to have higher rates of abdominal fat and insulin resistance than men [38]. These biological risk factors are associated with a higher risk of T2DM [38]. Furthermore, hypertension was significantly associated with pre-diabetes in the current study in agreement with report elsewhere in SSA [35]. A study in Nigeria by Okpechi et al. [39] showed a high prevalence of hypertension in SSA. Many complex factors have been identified as possible reasons for this, including high salt intake, low physical activity, and genetic vulnerability to high blood pressure [39]. Therefore, these high-risk groups should be targeted for lifestyle education, particularly the need for self-monitoring of body weight and periodic checks on fasting blood glucose. The outpatient departments in this setting need to include these clinical checks and lifestyle education in their routine services.

Type 2 diabetes mellitus was associated with advancing age, consistent with previous reports in SSA [34–36]. Surprisingly, this association did not reach the level of statistical significance, probably due to the small number of T2DM cases. Also, the highest glucose concentration was found in subjects over the age of 45 years (45–64 years) comparable to findings from previous reports from South Africa [40] and Cameroon [41]. The significant gender differences observed in the prevalence rates for T2DM in the current study were consistent with findings in sub-Saharan Africa [42–44]. The wealth index was associated with elevated glucose levels in the current study, consistent with previous reports from Malawi [20] and elsewhere in Europe [45]. Most of the subjects with T2DM (39.3%) were significantly poorer compared with subjects with normal glucose metabolism (17.5%). The inequality observed in the gender distribution of the socio-economic variables in the current study was concerning. A significant proportion of diabetic women compared to diabetic men were poorly educated, unemployed and poor. This may possibly have been due to the gender division of labour in Swaziland. A greater proportion of women in Swaziland were vulnerable and excluded from strategic gender roles [19]. Therefore, measures to improve the socio-economic status of women are warranted in Swaziland to curb the growing diabetes in the kingdom.

### Strengths and limitations of the study

The major strength of this study was the fact that the diagnosis of T2DM was based on the analysis of the blood samples. This prevented recall bias associated with a self-reported diabetes status. To researchers’ knowledge, this study provided the first age-adjusted estimates of the prevalence of diabetes and pre-diabetes in Swaziland. These data provided a baseline upon which cost-effective and culturally acceptable intervention could be devised to address the growing burden of diabetes in Swaziland.

The major limitation of the present study was that the prevalence rates for abnormal glucose metabolism (pre-diabetes and T2DM) were determined based on the assumption that the participants who presented themselves had fasted for 8 h before screening and diagnosis. The use of glycated hemoglobin (HbA1c) would have improved the reliability of the data since the need for fasting would have been eliminated. Furthermore, the patients who presented themselves at the OPD in the hospital may not have been representative of the larger Swazi population. Therefore, extrapolating these findings to the general Swazi population should be done with caution. Lastly, some of the known risk factors of diabetes (such as genetic predisposition) were not investigated in this study.

The study’s findings highlighted the need for the Swazi government to adopt policies to reduce the burden of T2DM in the Kingdom through healthcare services, with a special focus on women. This is important if the kingdom hopes to achieve the ambitious Sustainable Development [46] Goal number three (target 3.4) of reducing the NCD pre-mature mortality by one-third by 2030.

## Conclusions

In conclusion, this study reported a higher prevalence of both T2DM, and pre-diabetes than previously reported among patients in this hospital. This study also found an increasing prevalence of T2DM and pre-diabetes with advancing age. Unfortunately, most patients with T2DM in this study were newly diagnosed for the disease. This indicated that the screening practices in this hospital were not effective. Therefore, it is suggested that a routine blood glucose test be incorporated into healthcare services at outpatient departments in this hospital. As demonstrated in previous studies in Swaziland and elsewhere, the findings affirmed the hypothesis that modifiable risk factors play important role in the rising prevalence of T2DM in developing countries. Hence, cost-effective and culturally acceptable health education measures are needed to promote a healthy lifestyle among patients in this setting to halt the rising burden of T2DM in Swaziland.

## Supplementary information

**Additional file 1: Supplementary file 1.** Crude and age-adjusted prevalence rates of pre-diabetes and type 2 diabetes mellitus.

## Data Availability

All data generated or analysed during this study are included in this published article [and its supplementary information files].

## Abbreviations

AGM: Abnormal Glucose Metabolism
IFG: Impaired Fasting Glucose
IGT: Impaired Glucose Tolerance
SSA: Sub-Saharan Africa
T2DM: Type 2 Diabetes Mellitus
