# Supplementary material for: Prevalence of abnormal glucose metabolism among adults attending an outpatient department at a tertiary referral hospital in Swaziland: a cross-sectional study
Source: BMC Public Health. 2020 Mar 26;20:392. doi: 10.1186/s12889-020-08489-9 (PMC7098143; doi:10.1186/s12889-020-08489-9)
Supplement: Supplementary file 1 — Additional file 1: Supplementary file 1. Crude and age-adjusted prevalence rates of pre-diabetes and type 2 diabetes mellitus. [file 12889_2020_8489_MOESM1_ESM.docx]

**Supplementary Table 1. Crude and age-adjusted prevalence rates of pre-diabetes and type 2 diabetes mellitus**

| **Age group (years)** | **n** | **Population %*** | **Pre-diabetes cases** | **T2DM cases** | **Pre-diabetes Crude Prevalence** | **T2DM crude prevalence** | **Age- adjusted pre-diabetes prevalence** | **Age-adjusted T2DM prevalence** |
| --- | --- | --- | --- | --- | --- | --- | --- | --- |
| **Men** | | | | | | | | |
| 15-24 | 49 | 23.0 | 2 | 0 | 0.5 | 0.0 | 0.9 | 0.0 |
| 25-34 | 63 | 11.5 | 5 | 2 | 1.3 | 0.5 | 0.9 | 0.4 |
| 35-44 | 40 | 7.0 | 0 | 2 | 0.0 | 0.5 | 0.0 | 0.4 |
| 45-54 | 13 | 5.3 | 4 | 0 | 1.0 | 0.0 | 1.6 | 0.0 |
| 55-64 | 17 | 4.2 | 3 | 1 | 0.8 | 0.3 | 0.7 | 0.3 |
| 65+ | 15 | 3.0 | 0 | 1 | 0.0 | 0.3 | 0.0 | 0.2 |
| **Sub-Total** | **197** |  | **14** | **6** | **3.6** | **1.6** | **4.1** | **1.2** |
| **Women** | | | | | | | | |
| 15-24 | 42 | 21.3 | 2 | 4 | 0.5 | 1.0 | 1.0 | 2.0 |
| 25-34 | 45 | 12.3 | 0 | 3 | 0.0 | 0.8 | 0.0 | 0.8 |
| 35-44 | 44 | 8.7 | 1 | 5 | 0.2 | 1.3 | 0.2 | 1.0 |
| 45-54 | 27 | 6.8 | 2 | 5 | 0.5 | 1.3 | 0.5 | 1.3 |
| 55-64 | 14 | 4.6 | 4 | 2 | 1.0 | 0.5 | 1.3 | 0.7 |
| 65+ | 16 | 4.7 | 2 | 3 | 0.5 | 0.8 | 0.6 | 0.9 |
| **Sub-Total** | **188** |  | **11** | **22** | **2.7** | **5.7** | **3.6** | **6.6** |
| n, Sample size  *Age standardization were based on national 2006/07 Demographic and Health Survey | | | | | | |  |  |
